# Postweaning Grouping as a Strategy to Reduce Singly Housed Male Mice

**DOI:** 10.3390/ani10112135

**Published:** 2020-11-17

**Authors:** Roger Grífols, Carolina Zamora, Iván Ortega-Saez, Garikoitz Azkona

**Affiliations:** 1 Charles River Laboratories in PRBB, Doctor Aiguader, 88, 08003 Barcelona, Spain; rgrifols.extern@prbb.org (R.G.); czamora.extern@prbb.org (C.Z.); 2 Parc de Recerca Biomèdica de Barcelona (PRBB) Animal Facility, Doctor Aiguader, 88, 08003 Barcelona, Spain; ivanortegasaez@ub.edu; 3 Department of Basic Psychological Processes and Their Development, School of Psychology, Euskal Herriko Unibertsitatea (UPV/EHU), Tolosa Hiribidea, 70, 20018 Donostia, Spain

**Keywords:** juvenile, pubescent, aggressiveness, testosterone, mouse

## Abstract

**Simple Summary:**

It is important to raise laboratory mice in stable groups. However, sometimes we can find litters with only one male. Unfortunately, age is a factor to consider when grouping a newly weaned mouse with other males due to inter-male aggressiveness. Our results showed that CD1 and SCID Beige newly weaned males can be safely grouped with juvenile and pubescent mice. However, only juvenile C57BL/6J will accept a newly weaned mouse as a new member of the group. This strategy could be helpful to reduce the number of singly housed male mice used for scientific purposes.

**Abstract:**

Rearing laboratory mice in groups is important since social isolation after weaning induces brain alterations, which entails behavioral abnormalities in adulthood. Age is an important factor when grouping males of different litters due to inter-male aggressiveness. The aim of this study was to determine whether newly weaned mice could safely be grouped with late juvenile or early and late pubescent mice, and whether cage cleaning, the number of the hosting group members and testosterone plasma levels have any influence. Newly weaned C57BL/6J, CD1, and SCID Beige male mice were systematically grouped with same strain late juvenile, early or late pubescent male mice in clean or dirty cages of 1, 2 or 3 hosting members. We also analyzed plasma testosterone levels at different postnatal days. Our result showed that only strain and hosting male’s age influence agonistic behavior toward newly weaned mice. Thus, in order not to house a recently weaned male alone, we would recommend grouping it with late juvenile same strain mice in all studied strains. In the same way, CD1 and SCID Beige pubescent mice will admit a newly weaned mouse in their group. However, we would not recommend grouping newly weaned and pubescent C57BL/6J males.

## 1. Introduction

The mouse (*Mus musculus*) is a sociable and hierarchical species that in nature forms territories, which are inhabited by a small population formed by a dominant male, several females with their young, and juvenile mice. The size of the territory depends on the availability of food and the density of the population. Adult males can remain in the parental territory or disperse, depending on the density of the population and the aggressiveness of each individual [1]. The alpha male usually presents aggressiveness for dominance against subordinate males of the same social nucleus and territorial aggressiveness against intrusive males [2,3]. In laboratory, mice have been bred, and in many cases inbred, with poor stimuli and in restrictive environments [4]. Domestication and artificial selection have altered certain behavior patterns, such as defensive behaviors [5], while others remain intact, such as nest building [6] or grooming [7].

Laboratory mice were weaned at, approximately, postnatal day 21 (PND21) and separated from their mothers to form groups of 2 to 5 members of the same sex and strain, which favors the affiliate relationships between individuals of the same group [8]. Keeping mice in a group is important, since social isolation after weaning produces neurochemical alterations [8,9,10] and also alterations in brain connectivity [11], which entails behavioral alterations in adulthood [8]. In fact, socially isolated raised mice have been used to study various behavioral phenotypes during juvenile development, including the behavior type of depression and anxiety [12,13,14], and social and cognitive deficits [10,13].

As mentioned above, laboratory mice are often weaned into single-sex groups. However, sometimes, we can find litters with only one male or female. In the case of females, there is no major problem, since we can group the weaned female with older females of the same strain. The group of females usually welcomes the new member of the group without major problems. This is because females maintain affiliation relationships with the rest of their partners, building nests and sharing the breeding in pairs or groups of females throughout their lives [15]. In the case of males, age is a factor to consider. The onset of agonistic and aggressive behaviors during adolescence period (PND21 to PND60) is essential for the further development of adequate adult social behaviors [16]. In the juvenile stage, which goes from PND21 to PND34, rodents establish relationships between siblings that probably promote subsequent behavioral flexibility facilitating the integrity of neuronal circuits [17,18]. At puberty, 35–54 days of life, sexual differences develop including physiological mechanisms and characteristics of adult behavior [19]. Presumably, at this time they begin to show territorial behavior accompanied by dominant–subordinate relations, even among brothers of the same litter [20]. Different studies have analyzed whether adolescent male grouping can reduce the development of aggression between cage members with ambiguous results and more focused in CD1 strain [21,22,23,24].

In 2017, we started grouping newly weaned males of different strains, including genetically modified mice, with males of the same strain that were not older than one week. Thus, 21-day-old animals (newly weaned) were introduced in cages where 28-day-old males were living (early juvenile). This strategy allowed us to reduce by 31.5% the number of individualized males in a year, without increasing the number of wounds caused by fights, an indirect indicator of aggressiveness [25].

The objective of the study was to determine whether newly weaned mice can be grouped with late juvenile or early or late pubertal stage mice without observing agonistic behaviors, and whether cage cleaning, number of the hosting group members, and testosterone plasma levels influence it.

## 2. Materials and Methods

### 2.1. Animals

C57BL/6J, Crl:CD1(ICR) (CD1), and CB17.Cg-Prkdc < scid > Lyst < bg-J >/Crl (SCID Beige) male mice were born in our specific pathogen-free (SPF) [26] breeding zone. Mice of our colonies were socially housed, with up to four male and five female mice, in 1145T (403 × 165 × 174 mm; 435 cm^2^ floor area; Tecniplast) pressurized individually ventilated (PIV) cages (70 air changes/h). We used black poplar/aspen shavings (Lignocel Selectfine; Rettenmaier Ibérica S.L.) as litter, two sheets of tissue (Tork^®^; Essity Spain S.L., Alcobendas, Spain) as nesting material irradiated by Ionisos Iberica (Cuenca, Spain) and an in-house autoclaved cardboard cylinder (12.5 × 9 × 0.5 cm; Sodispan Research S.L., Coslada, Spain) as enrichment [27]. Once a week, mice, together with their nest, were transferred to clean cages picking them up by holding the base of their tails [23,28]. If the nest was dirty or it did not have enough material, new irradiated tissue was added. In the same way, if the cardboard was broken, a new one was provided. Mice had ad libitum access to water and diet (irradiated special diet services RM1 up to nine weeks of age, and RM3 for breeding pairs and young mice until 9 weeks old). Rooms were maintained under standard environmental conditions (humidity: 55 ± 10%; temperature: 20–24 °C) and a 12 h light/dark cycle (lights on at 8:00 a.m.). Animals were monitored every day. Animal care and use program was approved by Parc de Recerca Biomèdica de Barcelona (PRBB)—Ethics Committee and accredited by AAALAC International, following European (2010/63/UE) and Spanish (RD 53/2013) regulations.

### 2.2. Grouping Experiment

#### 2.2.1. Experimental Groups

We analyzed three factors that can influence aggressiveness towards the new member of the group. (1) Age of mice of the receiving/hosting group; late juvenile (31–34 days), early pubertal (35–41 days) or late pubertal (42–54 days) stage [8]. (2) Number of members—one, two or three members (1M, 2M or 3M)—of the hosting cage. Thus, a newly weaned male was introduced with one (1M), two (2M) or three (3M) late juvenile or early or late pubescent male mice from another litter. Two or three siblings composed 2M or 3M cages. (3) Cage cleaning—half of the weaned mice were introduced in clean cages, with clean shavings, cardboard, and nesting material. Dirty cages—the other half were introduced in cages where hosting group members were already living for a week. Thus, dirty cages were changed the first time after two weeks. Our results from a previous study indicated that in these conditions—PIV cages with a constant air flow of 70 air changes/h, poplar as bedding material and a maximum of 5 animals per cage—ammonia and carbon dioxide levels were within the desired range over two weeks [29]. After this first two weeks, animals were changed every week. Note, when the hosting group was composed by one member (1M), peers were removed and a recently weaned mouse was introduced to the cage.

In this experiment, a total of 270 newly weaned (n = 90 per strain), 540 late juvenile (n = 180 per strain), 540 early pubertal (n = 180 per strain), and 540 late pubertal (n = 180 per strain) male mice were used. Each newly weaned mouse was randomly housed in one of the above-mentioned conditions (5 cages per condition). The experimental group description is indicated in (Table 1).

#### 2.2.2. Experimental procedure

A newly weaned male was randomly introduced in a clean or dirty cage with one (1M), two (2M) or three (3M) late juvenile, early pubescent or late pubescent males at the start of the day (8–9 a.m.) by a female caretaker. Animals were monitored every 30 min during the first eight hours. Then, all cages were monitored every day to check animals’ physical condition until the new member reached 8 weeks of age (adult) by a male technician (R.G.) or animal welfare officer (I.O.) who participated in the study.

In addition to the general physical condition, we checked wounds due to fights, defined as a complete break in the continuity of the epithelium, as indicative of aggression. Animals were handled, the skin was inspected to determine the presence of wounds, and they were assessed according to a numeric grading system by a qualified male technician or animal welfare officer (Table 2) [30].

If a member of a cage presented mild wounds, a new cardboard cylinder was introduced as environmental enrichment in order to reduce aggressiveness [27]. If the aggressions persisted for the next 24 h, or wounds were classified as moderate or severe, the animal was singly housed. If in three cages of a defined condition we observed moderate or severe wounds, we concluded that under that condition, newly weaned males could not be grouped.

Animals became part of our colony once the experiment was finished.

### 2.3. Testosterone Determination

#### 2.3.1. Experimental Groups

A different group of animals was used in this experiment, in which we took blood to determine plasma testosterone levels from PND21, 28, 35, 42, and 60 mice. We analyzed ten animals per age and strain, thus a total of 150 samples/animals.

#### 2.3.2. Experimental procedure

All samples were collected between 10:00 h and 11:00 h. Animals were transferred to a laboratory one hour prior the extraction. Afterwards, animals were anesthetized with sodium pentobarbital (50 mg/kg) (Vetoquinol S.A., Madrid, Spain) and blood was drawn by cardiac puncture, in order to obtain the necessary sample quantity. Animals were killed by cervical dislocation. Blood was collected in Microvette^®^ 200Z (Sarstedt AG, Nümbrecht, Germany), centrifuged for 10 min at 1600× *g* and stored at −20 °C. Testosterone levels (ng/mL) were determined using the commercial “DEMEDITEC Testosterone rat/mouse ELISA” kit (Demeditec Diagnostics GmbH, Kiel, Germany).

### 2.4. Statistical Analysis

Estimation of the required sample size for the grouping experiment was carried out using the epidemiological program Win Episcope 2.0 (Zaragoza, Spain) [31], based on the results of our previous work in which the maximum prevalence of inter-male aggressiveness was 1.9% [25]. The results of these experiments were analyzed by the hosting group members’ age period with a chi-square test of independence to examine the relation among cleaning, the number of hosting members, and wound due to fights (IBM SPSS Statistics 26, Armonk, USA). Data are presented as total number of wounds due to fights in each condition. Testosterone plasma levels group comparison was performed using a two-way ANOVA, followed by Bonferroni’s post hoc test (6.01, GraphPad Software, Inc., San Diego, CA, USA). Data are presented as group mean ± standard error of the mean (SEM). In both statistical studies, values of *p* < 0.05 were considered statistically significant (95% confidence).

### 2.5. Ethical Approval

The Catalan Government and PRBB Ethics Committees approved the experimental protocol (DAAM 9103 and 10576). The results are described in accordance with the ARRIVE guidelines [32].

## 3. Results

### 3.1. Newly Weaned Mice Can Be Safely Grouped with Late Juvenile Males

**Figure 1:**
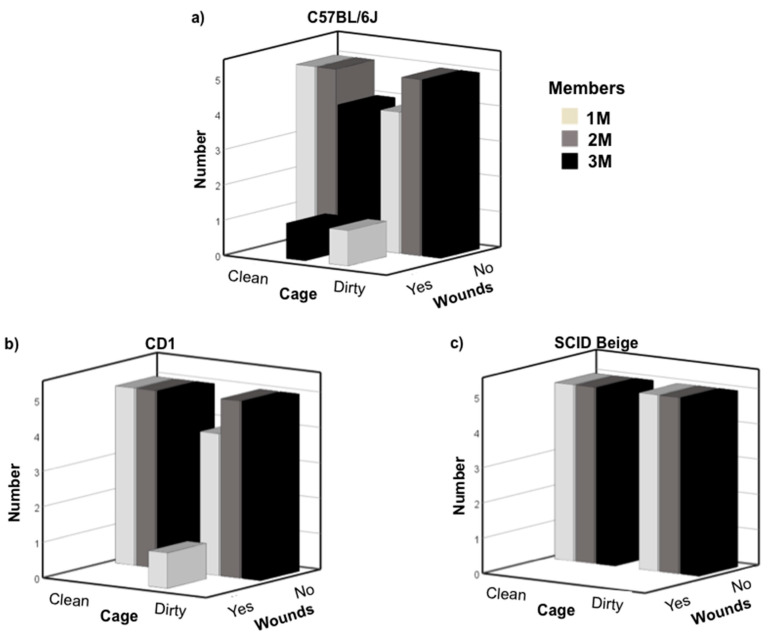
Number of cages in which wound due to fights were observed; (**a**) C57BL/6J (**b**) CD1 and (**c**) SCID Beige newly weaned mice after grouping with late juvenile male mice. X-axis shows the condition of the cage, clean or dirty. Z-axis shows whether there were or were not wounds due to fights. White bars indicate cages with one member; grey bars two members and black bar three members’ cages. A total of 30 cages per strain were analyzed, five cages per condition.

Newly weaned healthy male mice were grouped with late juvenile males of the same strain in the conditions explained above. The average age of juvenile males was 33.03 ± 0.22 days for C57BL6/J, 33.3 ± 0.27 for CD1 and 32.9 ± 0.23 for SCID Beige mice. The chi-square test of independence showed that there was no relationship among strain, cage condition, number of hosting group members, and wounds due to fights—X2 (2, N = 90) = 2.069, *p* = 0.355; Cramer’s V = 0.152. One newly weaned C57BL/6J grouped in a 3M clean cage, and another one in a 1M dirty cage presented moderate wounds due to fights three and four days after grouping, respectively, and were singly housed. Thus, 6.6% (2/30) of the cages presented moderate levels of aggressiveness in the C57BL/6J strain (Figure 1a). In the CD1 strain, we observed a case (1/30; 3.3%) of mild wounds due to fights in a 1M clean cage two days after they were grouped. Cardboard was added to the cage and no more fights were observed after that (Figure 1b). Regarding SCID Beige mice, no wounds due to fights were observed during the 8 weeks of the experiment (Figure 1c).

### 3.2. C57BL/6J Early Pubescent Males Showed More Agonistic Behaviour towards Newly Weaned Mice

**Figure 2:**
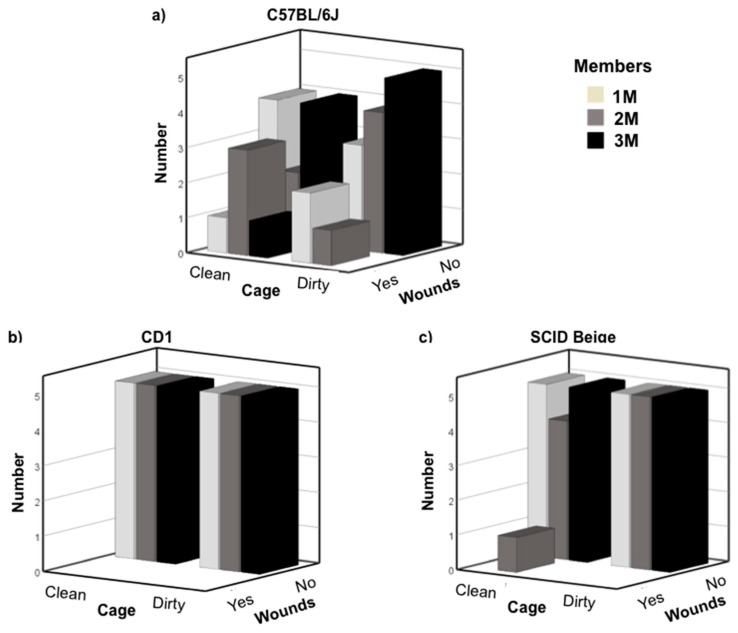
Number of cages in which wound due to fights were observed: (**a**) C57BL/6J, (**b**) CD1, and (**c**) SCID Beige newly weaned mice after grouping with early pubescent male mice. X-axis shows the condition of the cage, clean or dirty. Z-axis shows whether there were or were no wounds due to fights. White bars indicate cages with one member, grey bars two members, and black bar three members’ cages. A total of 30 cages per strain were analyzed—five cages per condition.

Next, we grouped newly weaned mice with early pubescent males. The average age of early pubescent males was 38.12 ± 0.29 days for C57BL/6J, 38.13 ± 0.36 for CD1 and 37.75 ± 0.28 for SCID Beige mice. Chi-square test of independence showed that there was a significant moderate association between strain and wounds due to fights—X2 (2, N = 90) = 14.074, *p* = 0.001; Cramer’s V = 0.395. Cleaning and the number of hosting group members were not associated with aggressiveness. Analyzing the data regarding strains, C57BL/6J showed more fights than the other strains. We observed severe wounds due to fights the morning after the grouping in two newly weaned mice grouped in 1M and 2M clean cages, and in a 2M dirty cage. These three newly weaned animals had to be sacrificed. In addition, five other newly weaned mice presented moderate wounds due to fights (two grouped in 2M clean cages, one in 3M clean cage, and two grouped in 1M dirty cages) and were singly housed. In total, we observed eight moderate-severe cases of wounds due to fights in the 30 analyzed cages (8/30; 26.6%) in the C57BL/6J strain. One experimental condition (2M clean cage) reached the maximum of three cages to conclude that under these conditions newly weaned males could not be grouped (Figure 2a). No fights were observed in CD1 mice (Figure 2b), but a newly weaned SCID Beige male grouped in a 2M clean cage presented mild wounds due to fights the day after grouping (1/30; 3.3%). Cardboard was added to the cage, and no more fights were observed (Figure 2c).

### 3.3. C57BL/6J Late Pubescent Males Showed More Agonistic Behaviour towards Newly Weaned Mice

**Figure 3:**
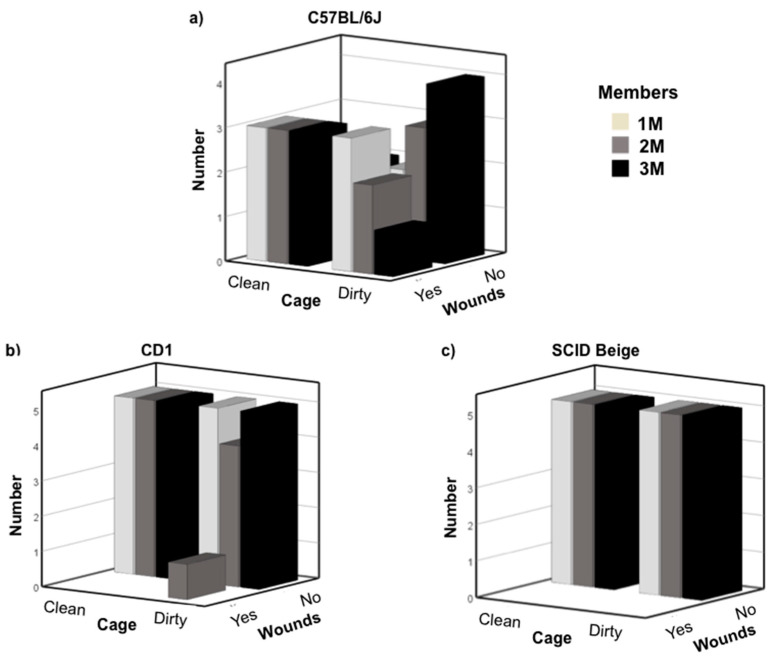
Number of cages in which wound due to fights were observed: (**a**) C57BL/6J (**b**) CD1, and (**c**) SCID Beige newly weaned mice after grouping with late pubescent male mice. X-axis shows the condition of the cage, clean or dirty. Z-axis shows whether there were or were no wounds due to fights. White bars indicate cages with one member; grey bars two members and black bar three members’ cages. A total of 30 cages per strain were analyzed—five cages per condition.

Finally, we grouped newly weaned males with late pubescent males. The average age of late pubertal stage males was 44.6 ± 0.32 days for C57BL/6J, 44.45 ± 0.37 for CD1, and 44.06 ± 0.36 for SCID Beige mice. Chi-square test of independence showed that there was a significant and relatively strong association between strain and wounds due to fights—X2 (2, N = 90) = 32.078, *p* = 0.000; Cramer’s V = 0.597. No association was observed between cleaning and the number of hosting group members with wounds due to fights. C57BL/6J mice showed more agonistic behaviors than the other strains. We found four weaned animals dead the morning after they were grouped (one in each of the following conditions—1M clean cage and 1M, 2M, and 3M dirty cages) and one with severe wounds due to fights that had to be sacrificed that was grouped in a 1M dirty cage. In addition, the day after grouping we observed moderate wounds due to fights in two 1M, two 2M, and three 3M clean cages and one in each of 1M, 2M, and 3M dirty cage conditions. These ten newly weaned mice had to be singly housed. In total, 50% (15/30) of the studied cages in the C57BL/6J strain presented wounds due to fights that were classified as moderate or severe. Four experimental conditions (1M, 2M, and 3M clean cage and 1M dirty cage) reached the maximum of three cages to conclude that under these conditions newly weaned males could not be grouped (Figure 3a). Regarding CD1 mice, we observed moderate wounds due to fights in a newly weaned mouse (1/30; 3.3%) grouped in a 2M dirty cage the day after grouping and singly housed (Figure 3b), but we did not observe any fights in SCID Beige mice (Figure 3c).

We want to note that no wounds to fights were observed in the late juvenile, early pubescent or late pubescent males, indicating that the entry of a new member does not increase the aggressiveness among the members of the hosting group.

### 3.4. No Differences in Testosterone Plasma Levels among Strains

**Figure 4:**
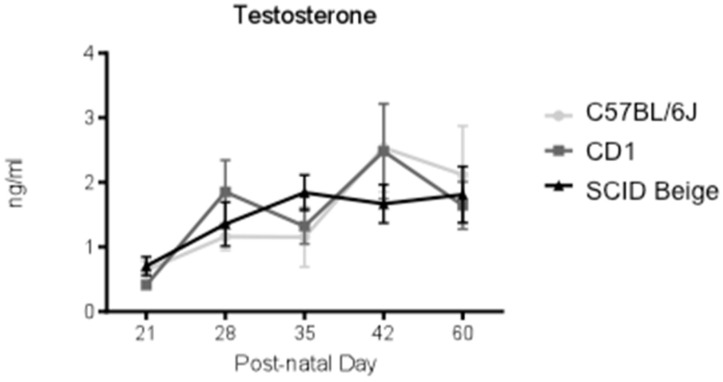
C57BL/6J, CD1, and SCID Beige testosterone plasma level (ng/mL) at different postnatal days. Data are expressed as mean ± SEM. We analyzed 10 animals per strain and age.

In order to determine plasma testosterone levels during juvenile and pubertal periods, blood samples were collected at different time points (21, 28, 35, 42, and 60 days). The results showed that testosterone levels increased with age—F_(4, 135)_ = 6.1; *p* = 0.0002 (Age effect). However, our results did not show statistically different testosterone plasma levels among the strains for any of the studied time points (Figure 4).

## 4. Discussion

We systematically introduced different newly weaned male mice into clean or dirty cages housed by one, two or three adolescent males of different age intervals to determine whether weaned mice can be grouped with pre-adult males in order to have a strategy to reduce the number of singly house laboratory male mice.

Given the large number of available strains and the phenotypic variability among them [33], we chose three of the most used strains in biomedical research. C57BL/6J inbred mice are the most used in behavioral studies and the generation of genetically modified models [34]; outbred CD1 mice are the most commonly used in toxicology studies [23], and SCID Beige immunodeficient mice are widely used in xenograft studies in oncology [35].

Our results showed that there were marked differences among strains, with C57BL/6J males being more aggressive toward newly weaned individuals. Regarding age periods, late juvenile C57BL/6J mice showed low levels of aggression (only two cases of moderate wound due to fights). However, pubescent, both early and late pubertal stage, males showed much more aggressiveness towards the newly weaned mice than the rest of the strains. In fact, eight newly weaned C57BL/6J mice were found dead and fifteen had to be singly housed the morning after grouping with pubescent male mice. This result indicates that moderate and severe wounds due to fights occurred during the first dark phase of the light/dark cycle, since we never observed fights during the eight hours of observation after the grouping. Although mice are nocturnal species [36], we did not expect, when designing the study, such a large difference between cycles. Thus, a major limitation of this study is our technical inability to monitor animals during the dark phase of the cycle. Overall, our results are in line with a previous study indicating that age is an important factor in inter-male aggressiveness, since early weaning reduced aggression-related injuries in this strain [37]. However, other studies reported that C57BL/6J adolescent males showed higher levels of sociability in different behavioral tests [38,39,40]. These studies measured sociability using a social choice paradigm conducted in a three-chamber apparatus or social interaction tests in a new cage, whereas we evaluated wounds due to fights assuming they were a result of aggression in their own home cage. This different result could be explained because intra-group and extra-group aggressions are not necessarily related and can have different underlying motivational states and consequences [41,42]. Nevertheless, adult C57BL/6J males are considered a low-prevalence aggressive mouse strain [43]. Regarding CD1 strain, adult males are described as aggressive and territorial [33,41,43,44]. Compared to the C57BL/6J strain, CD1 males showed a lower significant latency to first attack bout in an unconditioned aggression paradigm [45]. However, we observed very low levels of aggression between adolescent mice, in accordance with a recently published study [22]. To our knowledge, no previous work has analyzed aggressive behavior in SCID Beige male mice. We only observed mild wounds due to fights in a newly weaned mouse, indicative of very low inter-male pre-adult aggressiveness in this strain. Interestingly, mild wounds disappeared after introducing a new cardboard in the cage, in agreement with the report that environmental enrichment reduces aggressiveness [27]. In the case of moderate or severe wounds due to fights, we directly singly housed newly weaned mice because they needed veterinary treatment.

One factor that influences adult inter-male aggression is the number of males in the cage. It is widely accepted that three to five adult male laboratory mice groups result in more stable and less aggressive groups [22,24,44,46,47]. In fact, it has been proposed that group size should be optimized to three animals per cage [22,24]. Our statistical analysis did not show a hosting group size effect. This could be due to the fact that we did not exceed the recommended maximum limit of laboratory male mice housed in the same cage—a maximum of four males in an 1145T cage. However, 1M C57BL/6J late pubescent mice reached the maximum of three cages with moderate or severe wounds in both (dirty and clean) cleaning conditions and, interestingly, the number of cages in which we observed agonistic behaviors decreased as the number of hosting animals increased.

Cage cleaning has been identified as a cause of short-term increase in aggression in male mice [44]. We studied the influence of grouping newly weaned mice with juvenile or pubescent mice in two extreme conditions—clean and dirty cages. Our statistical results did not identify an effect of this factor. Previous studies reported contradictory results with a study recommending that cages should be completely cleaned and everything replaced [48], whereas others recommend to transfer the nesting material, because the used material contains pheromones with aggression-modulating properties that may be beneficial in groups where postcleaning aggression occurs [49] and does not negatively influence animal behavior in groups with low levels of aggression [44]. According to our statistical results, any of the aforementioned strategies regarding the cleaning of the cage can be effective. However, it should be considered that the number of cages in which agonistic behaviors of C57BL/6J pubescent mice toward newly weaned mice reached the maximum of three in four clean cages, and only in one dirty cage. As mentioned before, our husbandry protocol states that their nesting material always be transferred to the clean cage [23,28], and in our experience the prevalence of intra-male aggressiveness is very low (1.9%) [25].

Testosterone levels influence aggressive behaviors in adulthood during puberty [19]. We measured testosterone plasma levels at different time points during the adolescent period (from PDN21 to PDN60). Our results show that testosterone levels increase with age, as previously reported [50,51]. However, although we observed more antagonistic behaviors in C57BL/6J pubescent mice, the results did not show higher testosterone levels than CD1 and SCID Beige mice.

## 5. Conclusions

Our previous [25] and current results indicate that newly weaned lonely C57BL/6J, CD1, and SCID Beige mice can be grouped with juvenile mice of the same strain. They can be also be grouped with pubescent CD1 and SCID Beige male mice. However, we would not recommend grouping newly weaned and pubescent C57BL/6J males. Grouping newly weaned mice with juvenile mice of the same strain could be a helpful strategy to reduce the number of socially isolated male mice used for scientific purposes.

## Figures and Tables

**Table 1:** Experimental groups.

Age	Juvenil		Early Pubertal		Late Pubertal	
Cage Members	Clean	Dirty	Clean	Dirty	Clean	Dirty
1M	5	5	5	5	5	5
2M	5	5	5	5	5	5
3M	5	5	5	5	5	5

The age period of the hosting males is indicated in the first row and in the second row the cleaning conditions. The first column indicates the hosting group composition: 1, 2 or 3 members (Ms).

**Table 2:** Wound assessment metric.

Grade	Number of Puncture Wounds	Severity Classification
1	1–10	Mild
2	11–20	Moderate
3	≥21	Severe
